# The role of social media in long-running live events: The case of the Big Four fashion weeks dataset

**DOI:** 10.1016/j.dib.2021.106840

**Published:** 2021-02-12

**Authors:** Marco Brambilla, Alireza Javadian Sabet, Marjan Hosseini

**Affiliations:** a Dipartimento di Elettronica, Informazione e Bioingegneria, Politecnico di Milano, Via Giuseppe Ponzio, 34, I-20133 Milano, Italy; b Computer Science and Engineering Department, University of Connecticut, 369 Fairfield Way, Storrs, CT 06268, United States

**Keywords:** Social media, Instagram, Fashion, Live events, Social network, Brand, Popularity

## Abstract

The advent of social media platforms has caused many changes in humans’ daily lifestyle. One of the most significant changes is the way in which people participate in social and cultural events. Users' participation in social media platforms is continuously increasing. This has provided brands with new opportunities such as enhancing brand influence and understanding online users' reactions through user-generated content (UGC) analysis.

We provide and describe a large-scale hashtag-based dataset of social media posts published on Instagram about the Big Four international fashion weeks in New York, Paris, Milan, and London. The dataset provides the data of the 2018 events and has a periodic and well-established structure.

Moreover, we designed a two-stage platform for collecting such large-scale datasets related to long-running events based on relevant hashtags: In the first stage, the platform extracts all the posts, and in the second stage, it extracts the information about the authors of the posts.

## Specifications Table

**Table:** 

Subject	Human-Computer Interaction
Specific subject area	Social media user's behaviour on long-running live events. The case of international Fashion Weeks events.
Type of data	Table
How data were acquired	We collected publicly available posts and the post's user profile on Instagram regarding Fashion Weeks events using Instagram's API.
Data format	Raw
Parameters for data collection	We used the hashtags presented in Table 1 as the seeds to query Instagram's API.
Description of data collection	We found the events' most used hashtags by manually exploring Instagram's search function and other online resources as the hashtag seeds (presented in Table 1). Then we requested Instagram's API to collect the posts containing those hashtags. After applying the cleaning steps, by using Instagram's API, we requested to collect the user's profile who authored those posts (if their profile were publicly available).
Data source location	Instagram posts generated during Fashion Week events from all around the world.
Data accessibility	Repository name: Harvard Dataverse Data identification number: UNF:6:68IyOS0ZSuPmLjTEHLzK2Q== Direct URL to data: https://dataverse.harvard.edu/citation?persistentId=doi:10.7910/DVN/8BNXES[1]

## Value of the Data

• Due to the information cascade on social media (SM) [2,3], the information obtained from the well-established events that are covered by SM can be useful for brands and businesses to identify various communities preferences, and consequently, promote users’ engagement [4].
• The presented dataset can be used as a benchmark dataset for brands to promote brand awareness and improve the quality of customer relationship management (CRM) by way of discovering users’ online preferences toward brands, products and different topics.
• It can be leveraged to design context-aware recommender systems [5] in order to recommend the most suitable product according to users’ preferences.
• Event organizers including municipalities are the other beneficiaries who can make use of this information for logistic purposes so as to improve the quality of urban life.

## 1 Data Description

We found the events' most-used hashtags by manually exploring Instagram's search function and other online resources as the hashtag seeds (presented in Table 1).

**Table 1:** List of hashtags used for data collection.

City	Hashtags
Milan	#milanofashionweek2018, #milanfashionweekss18, #milanfashionweek, #mfwp, #milanfashionweek18, #mfw, #milanfashionweek2018, #milanofashionweek18, #cameramoda, #milanofashionweek, #mfwreporter, #mfwstreetstyle, #mfwadventures, #milanfw2018, #milanfw18, #milanofw18, #milanofw, #mfw2018, #mfwss2018, #mfwaw18, #wmfw, #mfwss18, #mfwf, #mfwfw18, #milanfw, #mfwlive, #mfw18
Paris	#pfwmenswear, #parisfashionweekmens, #pfw_post, #pfwstreetstyle, #pfwss18, #pfw2018, #pfwss2018, #pfwfw18, #pfwcouture, #parisfw18, #parisfw, #parisfwss18, #parisfashionweek, #parisfashionweek2018, #pfw18, #parisfashionweekscenes, #pfwaw18, #pfw, #pfwlive, #pfwfashionweek
London	#londonfashionweekmens, #londonfashionweek18, #londonfashionweekmen, #lfww, #londonfashionblogger, #londonfashionweek, #londonfashionweek2018, #lfwmens, #londonfashion2018, #lfw18, #lfw2018, #lfwm, #lfwm2018, #londonfw18, #lfwmen, #lfashionweek, #londonfw, #lfw, #londonfashion
New York	#nyfashionweek, #newyorkfashionweek, #nyfwaw18, #nyfwcastings, #nyfwmodel, #newyorkcityfashionweek2018, #newyorkcityfashionweek, #nycfashionweek, #nyfw, #nycfashionweek2018, #nyfwkidsshows, #nyfw18, #nyfashionweek2018, #nyfw2018, #nyfw2018ss, #nyfwss18, #nyfwss, #nyfwstreetstyle, #nyfww, #nyfwblogger, #newyorkfashionweek2018, #nyfwmens, #nyfwm, #nyfw4all, #nyfwbridal

The dataset that we provide is composed of two comma-separated values CSV files: posts and user profiles authoring the posts. The resulting dataset comprises *905,726* posts and *171,078* correspondent unique user profiles. Details on their attributes are provided in the following lists:

## 2 Posts Dataset Columns and Descriptions

• **Post's PK**: ID of the post.
• **User's PK:** Anonymized ID of the post author.
• **Likes Count:** Total number of likes.
• **Comments Count:** Total number of comments.
• **Time_In:** 1 if the post was published in the target event period.
• **Time_Other:** 1 if the post was published in other events periods.
• **Time_None:** 1 if the post was published in none of the events periods.
• **Caption Length:** Number of characters in the post caption.
• **Hashtags Count:** Number of used hashtags in the post caption.
• **Event_Milan:** 1 if the post was about Milan FW.
• **Event_Paris:** 1 if the post was about Paris FW.
• **Event_London:** 1 if the post was about London FW.
• **Event_NewYork:** 1 if the post was about NY FW.
• **Brand_X:** We provided a Boolean column for each of the following 21 brands. Each of them can take 1 if the post caption contains their related hashtag. The covered brands are: Gucci, Chanel, Dior, Fendi, Burberry, D&G, Balenciaga, Versace, Prada, LouisVuitton, Tommy, Nike, Valentino, Adidas, Zara, CalvinKlein, VictoriaSecret, Miumiu, Bvlgari, H&M, Armani.

Due to copyright and privacy regulations by Instagram and posts authors, we solely publish the attributes that we prepared. However, it is possible to access the posts (if publicly available at the time of request) through the post's identifier (PK).

## 3 Users Dataset Columns and Descriptions

• **User's PK:** Anonymized ID of the user.
• **Event Posts Count:** Number of posts by the user in the dataset.
• **Event Likes_X:** Highest, Sum, Average, Median of likes of user's posts in the event.
• **Event Comments_X:** Highest, Sum, Average, Median of Comments of user's posts in the event.
• **Event Geo-tagged Percent:** The percentage of the user Geo posts.

In the following, we provide some descriptive statistics about the collected datasets.

### 3.1 Hashtags frequency

We investigated the hashtags mentioned in the *posts' caption* including hashtag seeds and the new ones. Next, we extracted unique hashtags and their usage percentage in the posts’ captions. In other words, we calculated the ratio of the number of posts containing a hashtag to the total number of posts for each hashtag. We found the most frequently used hashtags in the dataset. The total number of hashtags used in the posts and the unique ones are 13,880,586 and 476,907, respectively. Among the latter, only 69,353 (14.54%) have been used more than or equal to 10 times.

**Fig. 1:**
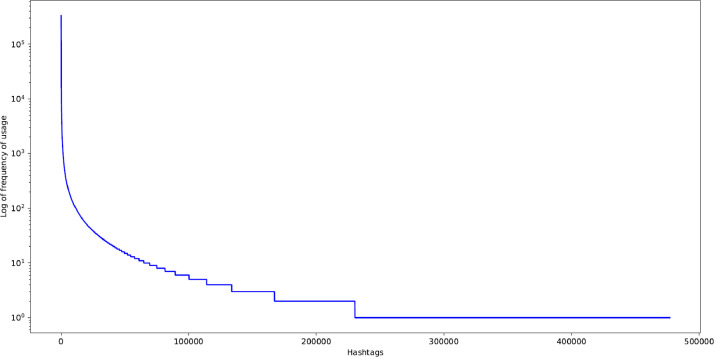
Posts' hashtag usage frequency. The x-axis lists the usage ranks of the hashtags, while the y-axis reports the logarithm of the frequency.

**Fig. 2:**
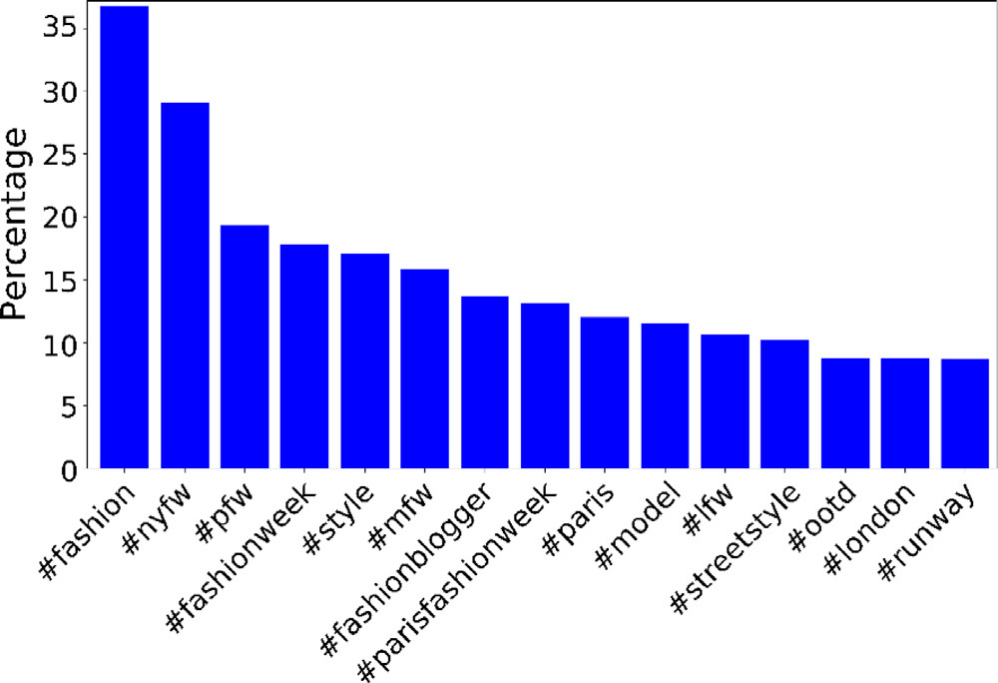
Top 15 most-used hashtags in the dataset. The x-axis lists hashtags ordered by their percentage of usage, while the y-axis reports the percentage of the posts to contain those hashtags.

Since the distribution of hashtags usage frequency is highly heavy-tailed, Fig. 1 presents it on a logarithmic scale. Fig. 2 depicts the top 15 most-used hashtags with their usage percentage.

### 3.2 Hashtag relevancy analysis

In order to inspect the extent to which posts are truly related to the event represented by the hashtags in their caption, it is possible to add four extra Boolean fields, namely *Milan, Paris, London*, and *New York* to each post. Their values represent if the post's caption contains at least one of the hashtags used for the data collection of that city.

**Fig. 3:**
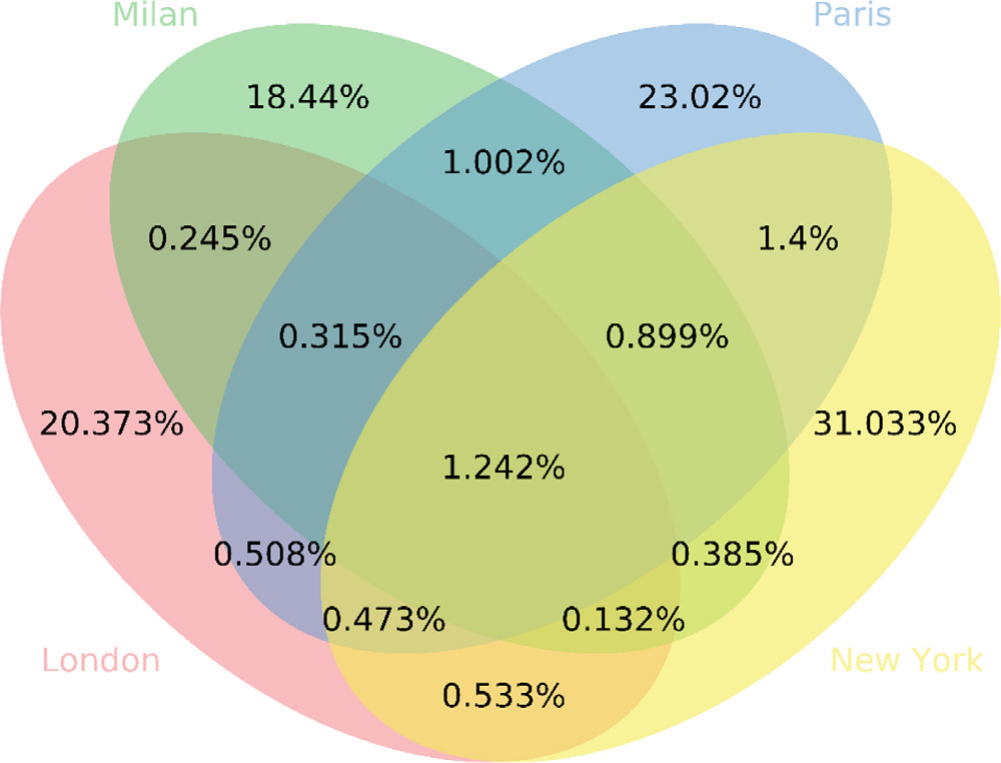
The Venn diagram represents the portion of dataset posts that contains hashtags of the different combinations of cities.

Subsequently, in order to depict the degree to which the posts of each city overlaps, we calculated the percentage of posts related to the cities. The Venn diagram in Fig. 3 presents all the possible logical states of the posts in relation to the cities.

### 3.3 User related statistics

Users who posted content targeting more than one city might have used a series of hashtags only for different reasons, such as increasing visibility. The distinguishable characteristics of the users who have used hashtags related to multiple events (cities) at the same time can be further analyzed. However, at this stage, we consider a rough estimation of three categories of the users according to their posting behaviour as follows:

• **Pure Content Generators:** The users who have always posted using hashtags related to only one city at a time (94.1% of the users).
• **Mixed Content Generators:** The users who have always posted using multiple event-related hashtags (1.86% of the users).
• **Pure and Mixed Content Generators:** The users who have both pure and mixed posts (3.23% of the users).

**Fig. 4:**
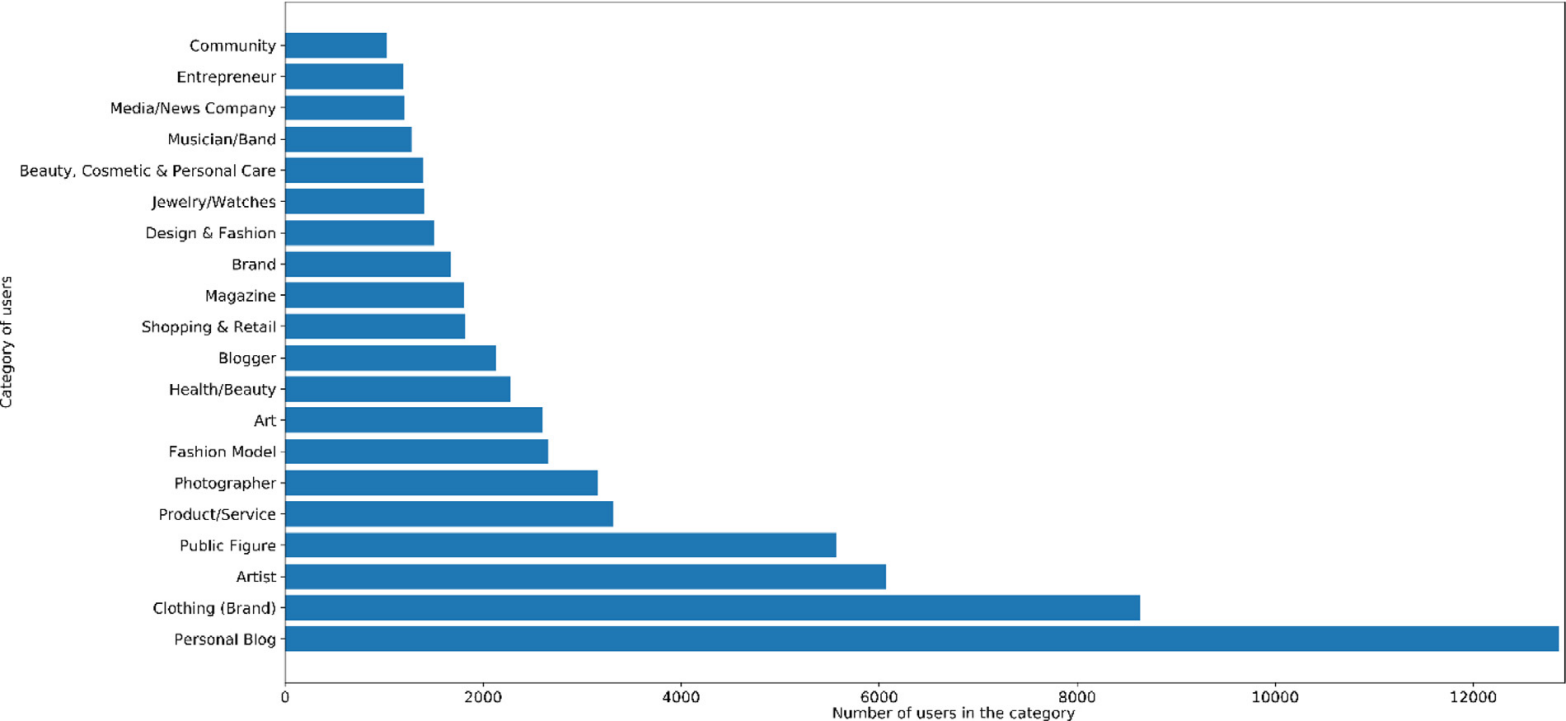
Top twenty categories of the users in the dataset.

Instagram users may provide some information in their profiles regarding their category. 53.2% of the user profiles in the dataset provided information regarding their category. Fig. 4 lists the top-twenty identified categories among the users.

**Fig. 5:**
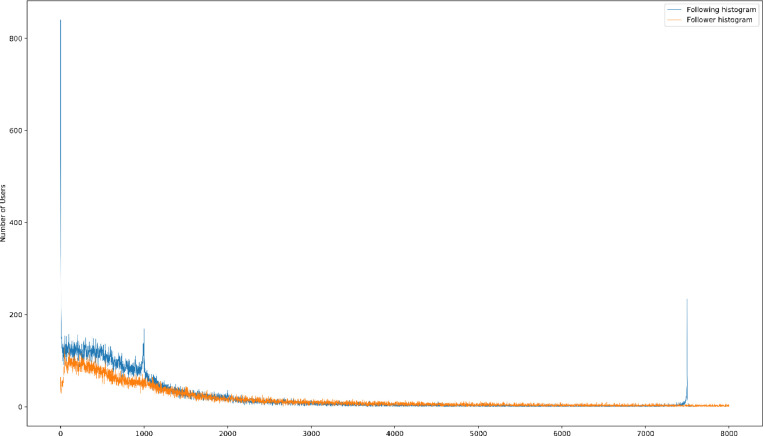
Histogram of the number of following in blue and followers in orange on x-axis both limited to 10,000 and the number of users with the corresponding numbers on the y-axis for the Instagram user's profile in the dataset.

With the aim to investigate users’ basic network, we plotted a histogram (presented in Fig. 5). The x-axis represents the following and followers count, and the y-axis represents the number of users that have such numbers as their followings and followers counts.

### 3.4 Temporal statistics

**Fig. 6:**
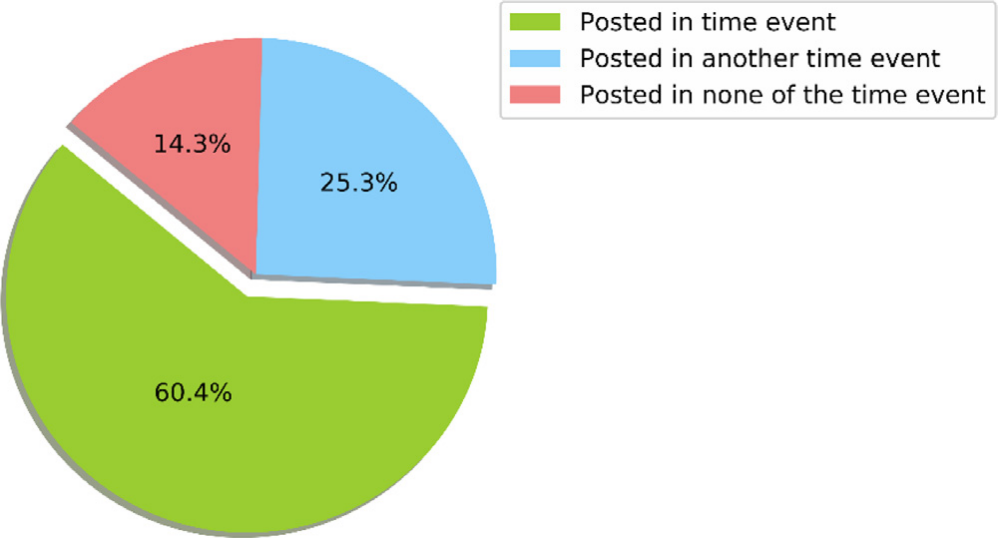
Dataset posts' timing according to the actual events calendar.

To investigate the information obtained from the posts regarding the date and time they were published, we provided additional temporal-related information for each post in three one-hot encodings, which determine whether the publishing time coincide with the actual event time in that particular city mentioned in the hashtag, or it happened during the event in other cities, or it was published outside all the events interval. We reported the categorization in Fig. 6.

### 3.5 Location related statistics

**Fig. 7:**
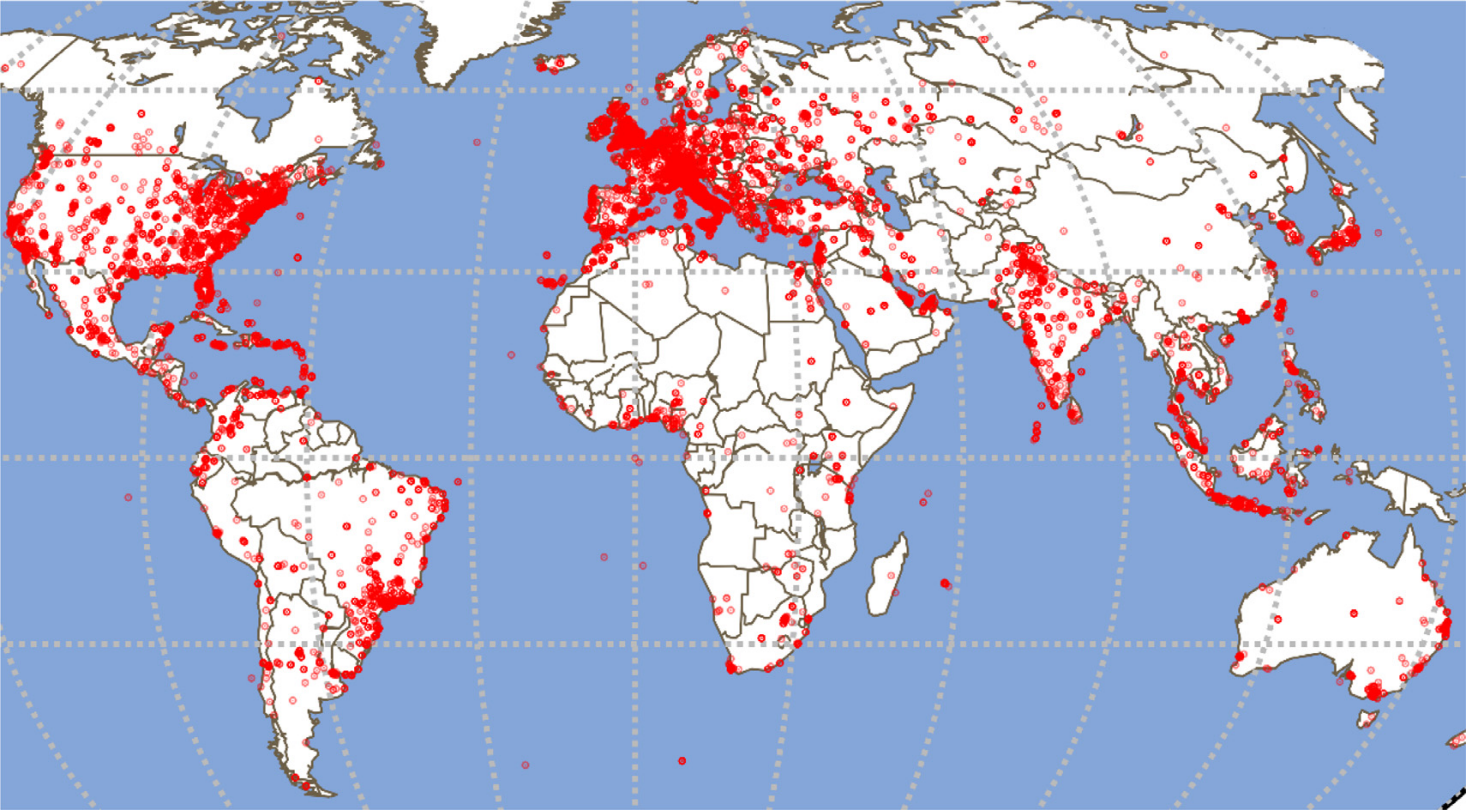
Geographical distribution of the Geo-located posts in the dataset.

**Fig. 8:**
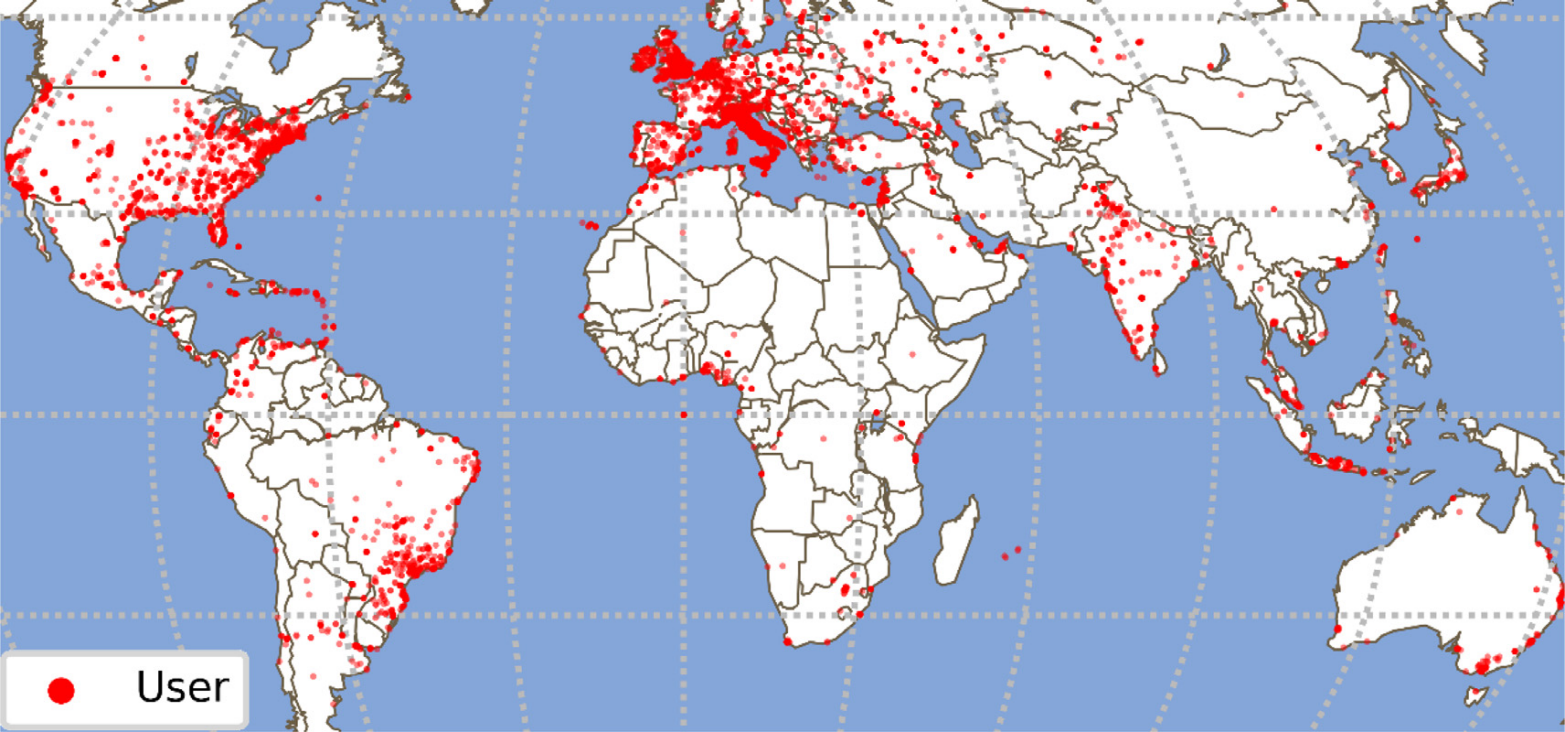
Geographical dispersion of the users' locations in the dataset.

Among 905,726 collected posts, 42.59% are geo-tagged. We report the posts spatial distribution employing these metadata in Fig. 7. The red dots indicate the location of the posts published for all cities. Furthermore, we depicted the users' geographical distribution in Fig. 8. The red dots in the map account for 53.16% of the users in the dataset for whom the location metadata was available at the time of data collection.

### 3.6 Brand related statistics

We identified the main brands in fashion week events gained more attention from the users i.e., more than 1,500 posts containing the hashtags related to those brands and reported them in Table 2.

**Table 2:** Top brands with more than 1,500 related posts (i.e., containing hashtags related to each brand) in the dataset.

Brand	Related posts Frequency	Brand	Related posts Frequency
Chanel	17,653	Zara	5,480
Gucci	17,234	Armani	4,957
Dior	15,370	Nike	4,733
Fendi	9,168	Tommy	4,695
Louis Vuitton	8,651	Victoria Secret	4,077
D&G	8,584	Adidas	3,512
Prada	8,154	CalvinKlein	3,016
Versace	7,297	H&M	2,585
Valentino	6,466	Miumiu	1,993
Balenciaga	5,938	Bvlgari	1,661
Burberry	5,762		

## 4 Experimental Design, Materials and Methods

We collected the presented dataset using Instagram API^1^ directly, since, to the best of our knowledge, there is no benchmark dataset regarding Big Four FWs. The data includes event-related posts and media shared on Instagram from January 1^st^, 2018 to March 11^th^, 2018 (five days before the first event i.e., London Fashion Week Men and five days after the last event i.e., Paris Fashion Week).

We discovered the events' most-used hashtags by way of manually exploring Instagram's search function and other online resources as the hashtag seeds (presented in Table 1). We collected over 3 million related public posts and the authors' profiles.

Unlike many other studies that collected posts of a few or specific types of users such as celebrities, we added diversity to the data by adopting a *hashtag-based* data collection approach.

• **Data Preparation:** We converted data from JSON format to CSV and we removed unnecessary attributes provided by Instagram's API.
• **Data Cleaning:** Due to the inherent noise in the collected data based on keyword search [6], we implemented the following data cleaning approaches.
  ○ Duplication removal is the process of removing duplicated posts, which are due to the collecting the posts that contain multiple hashtags of the hashtag seeds in their captions.
  ○ Field error removal eliminates the posts containing NaN values in their fields, which are generally as a result of API or network-related problems during the data collection stage.
  ○ Out of interest duration removal is necessary because the API had to inevitably crawl backward from the collection date, which accumulated many unwanted posts published in out-of-study dates.
  ○ Off-topic removal was applied to eliminate the posts which do not contain any of the initial hashtags for data collection. This type of posts is collected because in the hashtag-based search, the Instagram's API retrieves the posts even if the target hashtags exist in the posts' comments, and not necessarily just in the caption. Since the captions are created by the author, only the hashtags in this part should be considered.

## Ethics Statement

Data has been collected according to the data owner terms of service. The dataset described here is not publishing the actual content of the collected posts, and thus we are complying with the regulations provided by the platform owner.

## CRediT Author Statement

**Marco Brambilla:** Supervision, Funding acquisition, Conceptualization, Methodology, Validation, Investigation, Writing review & editing; **Alireza Javadian Sabet:** Conceptualization, Methodology, Software, Validation, Investigation, Data curtion, Writing original draft, Visualization; **Marjan Hosseini:** Software, Validation, Investigation, Data curtion, Writing original draft, Visualization.

## Declaration of Competing Interest

The authors declare no conflict of interest in this article.

## Footnotes

1 https://www.instagram.com/developer/.
